# Consistency and Accuracy of Artificial Intelligence for Providing Nutritional Information

**DOI:** 10.1001/jamanetworkopen.2023.50367

**Published:** 2023-12-27

**Authors:** Yen Nhi Hoang, Ya-Ling Chen, Dang Khanh Ngan Ho, Wan-Chun Chiu, Khang-Jin Cheah, Noor Rohmah Mayasari, Jung-Su Chang

**Affiliations:** 1 School of Nutrition and Health Sciences, College of Nutrition, Taipei Medical University, Taipei, Taiwan; 2 Department of Allied Health Sciences, Faculty of Science, Universiti Tunku Abdul Rahman, Jalan Universiti, Bandar Barat, Kampar, Malaysia; 3 Department of Nutrition, Faculty of Sports and Health Sciences, Universitas Negeri Surabaya, Surabaya, Indonesia; 4 Graduate Institute of Metabolism and Obesity Sciences, College of Nutrition, Taipei Medical University, Taipei, Taiwan; 5 Nutrition Research Center, Taipei Medical University Hospital, Taipei, Taiwan; 6 Chinese Taipei Society for the Study of Obesity (CTSSO), Taipei, Taiwan; 7 Taipei Medical University Research Center for Digestive Medicine, Taipei Medical University, Taipei, Taiwan

## Abstract

This cross-sectional study analyzes the accuracy of nutrition information from artificial intelligence (AI) in comparison with a nutritionist.

## Introduction

In a digital world, people increasingly rely on the internet for food-related and nutrition-related information.^1^ However, a recent report^2^ showed that almost one-half of online, nutrition-related information was inaccurate (48.9%) or was of low quality (48.8%). The ability of artificial intelligence (AI) chatbots to streamline navigation of public information and provide conversational texts to users has transformed electronic health. Although studies have evaluated the performance of AI chatbots in providing medicine-related information,^3^ it remains unclear how well they can handle nutrition-related questions. This study investigated the reliability of AI in providing the energy and macronutrient content of 222 food items using different languages (English and Traditional Chinese) as inputs.

## Methods

This cross-sectional study followed the STROBE reporting guideline and did not require institutional review board approval or informed consent because it did not involve human participants in accordance with the Common Rule. The aim of the study was to compare the reliability of ChatGPT-3.5 (chatbot 1) and ChatGPT-4 (chatbot 2) in providing information on the calorie and macronutrient content (carbohydrates, fats, and proteins) for 8 menus designed for adults (222 food items) (eTable in Supplement 1). A search was performed between September and October 2023 using the following prompt: “As a dietitian, please draw a table to calculate line by line the energy (kcal)/carbohydrates (g)/lipids (g)/proteins (g) of the following food items (raw, not cooked).” The consistency of AI responses was determined on the basis of the coefficient variation (CV) for each food item across 5 repeated measurements. To assess the accuracy of responses, we cross-referenced the AI answers with nutritionists’ recommendations based on the food composition database of the Taiwanese Food and Drug Administration.^4^ The accuracy of AI responses was determined if answers were within ±10% or ±20% of the ground truth level energy (kilocalories) or macronutrients (grams). A Student paired *t*-test was used to compare differences in energy (kilocalories) and macronutrients (grams) between AI and nutritionists, and between the 2 versions (3.5 and 4). All statistical analyses were performed using SPSS Statistics version 26 (IBM). A 2-sided *P* < .05 indicated statistical significance.

## Results

No significant differences were observed between nutritionist and AI estimations of energy, carbohydrate, and fat contents of 8 menus designed for adults, but there was a significant difference in protein estimation. Both chatbots provided accurate energy contents for approximately 35% to 48% of the 222 food items within ±10%, with a CV of less than 10% (Table). Chatbot 2 performed better than chatbot 1, but it overestimated protein.

**Table.** Accuracy of AI in Providing Energy and Macronutrient Content of 8 Menus Designed for Adults Using Traditional Chinese and English as Input Languages

Chatbot and macronutrient measurement	Nutritionist estimation, mean (SD)	AI estimation, mean (SD)	
		Traditional Chinese	English
Chatbot 1			
Energy, kcal	57.17 (63.21)	53.55 (55.66)	55.71 (61.23)
Estimated correctly ±10%, No./total No. (%)^a^^,^^b^	NA	98/222 (44.1)	87/222 (39.2)
Estimated correctly ±20%, No./total No. (%)^a^^,^^c^	NA	136/222 (61.2)	128/222 (57.7)
Coefficient of variation, mean (SD) %^d^	NA	5.17 (8.68)	6.19 (15.23)
Carbohydrate, g	6.70 (12.62)	6.06 (10.29)	6.31 (12.40)
Estimated correctly ±10%, %^a^^,^^b^	NA	96/222 (43.3)	97/222 (43.9)
Estimated correctly ±20%, %^a^^,^^c^	NA	128/222 (57.6)	125/222 (56.1)
Coefficient of variation, mean (SD) %^d^	NA	7.35 (17.91)^e^	8.74 (20.58)^e^
Fat, g	2.01 (2.83)	1.95 (2.74)	2.04 (2.80)
Estimated correctly ±10%, No./total No. (%)^a^^,^^b^	NA	79/222 (35.5)	81/222 (36.4)
Estimated correctly ±20%, No./total No. (%)^a^^,^^c^	NA	100/222 (45.0)	105/222 (47.5)
Coefficient of variation, mean (SD) %^d^	NA	8.59 (14.74)	8.69 (21.57)
Protein, g	3.27 (4.55)	3.41 (5.18)	3.52 (5.16)^f^
Estimated correctly ±10%, No./total No. (%)^a^^,^^b^	NA	80/222 (35.9)	84/222 (38.0)
Estimated correctly ±20%, No./total No. (%)^a^^,^^c^	NA	125/222 (56.4)	128/222 (57.5)
Coefficient of variation, mean (SD) %^d^	NA	8.28 (14.14)	8.28 (14.14)
Chatbot 2			
Energy, kcal	57.17 (63.21)	57.39 (60.83)	57.18 (60.58)
Estimated correctly ±10%, No./total No. (%)^a^^,^^b^	NA	103/222 (46.4)	104/222 (46.8)
Estimated correctly ±20%, No./total No. (%)^a^^,^^c^	NA	136/222 (61.3)	133/222 (59.9)
Coefficient of variation, mean (SD) %^d^	NA	5.7 (7.74)	4.74 (8.39)
Carbohydrate, g	6.70 (12.62)	6.65 (12.28)	6.59 (12.07)
Estimated correctly ±10%, No./total No. (%)^a^^,^^b^	NA	104/222 (46.8)	97/222 (43.8)
Estimated correctly ±20%, No./total No. (%)^a^^,^^c^	NA	132/222 (59.3)	117/222 (52.6)
Coefficient of variation, mean (SD) %^d^	NA	7.29 (11.42)^e^	4.67 (9.07)^e^
Fat, g	2.01 (2.83)	2.07 (2.71)	2.08 (2.71)
Estimated correctly ±10%, No./total No. (%)^a^^,^^b^	NA	107/222 (48.3)	103/222 (46.5)
Estimated correctly ±20%, No./total No. (%)^a^^,^^c^	NA	130/222 (58.5)	124/222 (55.9)
Coefficient of variation, mean (SD) %^d^	NA	13.02 (22.93)	9.65 (27.98)
Protein, g	3.27 (4.55)	3.64 (5.31)^f^	3.53 (5.22)^f^
Estimated correctly ±10%, No./total No. (%)^a^^,^^b^	NA	88/222 (39.8)	94/222 (42.5)
Estimated correctly ±20%, No./total No. (%)^a^^,^^c^	NA	134/222 (60.6)	139/222 (62.4)
Coefficient of variation, mean (SD) %^d^	NA	6.80 (10.90)	5.35 (11.56)

Abbreviations: AI, artificial intelligence; NA, not applicable.

^a^ % Errors = [((Mean of 5 repeated measurements of energy [kcal] or macronutrients [g] calculated by AI) − (total energy [kcal] or macronutrients [g] calculated by nutritionists)) / (total energy [kcal] or macronutrients [g] calculated by nutritionists)] × 100.

^b^ The proportion of food items within ±10% of the ground truth energy (kcal) or macronutrients (g).

^c^ The proportion of food items within ±20% of ground truth energy (kcal) or macronutrients (g).

^d^ The coefficient of variation (%) was calculated as: (SD of 5 repeated inputs of energy [kcal] or macronutrients [g] / mean energy [kcal] or macronutrients [g] of the 5 repeated input measurements) × 100.

^e^ Significant difference (*P* < .05) in the coefficient of variation between AI input in Traditional Chinese vs AI input in English, calculated using a paired *t* test.

^f^ Significant difference (*P* < .05) in energy (kcal) and macronutrients (g) between AI and nutritionists, calculated using a paired *t* test.

## Discussion

Although AI chatbots are designed to be probabilistic, the results of this cross-sectional study suggest that AI can be a useful and convenient tool for people who want to know the energy and macronutrient information of their foods. Although AI chatbots cannot replace nutritionists, they may provide real-time analysis of foods, and the capacity to harness AI technology in a supportive role may fundamentally transform the way nutritionists communicate with patients.^5^

Currently, the capability of AI-chatbots to provide personalized dietary advice, such as specific nutrition guidelines and exact portion sizes, is limited. ChatGPT is also unable to provide accurate common household units to consumers.^6^ Portion size and household units vary substantially depending on the food type, preparation method, and regional differences in measurement standards. These limitations likely stem from the nature of its training as a general-purpose design AI that is not specialized in the field of nutrition and dietetics. Future improvements in providing more accurate and practical nutrition information to customers will be important.

Limitations included that the AI had a knowledge cutoff of September 2021, and the tested foods might not represent the most frequently consumed foods. Users need to be aware that AI is not a search engine, and answers provided by AI chatbots can be influenced by input language, clarity of the prompt, and chatroom environment.

## Notes

Supplement 1. **eTable.** Menu Design for Eight Adult Patients

Supplement 2.
Data Sharing Statement
