# Supplementary material for: Consistency and Accuracy of Artificial Intelligence for Providing Nutritional Information
Source: JAMA Netw Open. 2023 Dec 27;6(12):e2350367. doi: 10.1001/jamanetworkopen.2023.50367 (PMC10753390; doi:10.1001/jamanetworkopen.2023.50367)
Supplement: Supplement 1. — eTable. Menu Design for Eight Adult Patients [file jamanetwopen-e2350367-s001.pdf]

Supplemental Online Content

Hoang YN, Chen YL, Ho DKN, et al. Consistency and accuracy of artificial intelligence for providing nutritional inofrmation. *JAMA Netw Open*. 2023;6(12):e2350367. doi:10.1001/jamanetworkopen.2023.50367

eTable. Menu Design for Eight Adult Patients

This supplemental material has been provided by the authors to give readers additional information about their work.

© 2023 Hoang YN et al. *JAMA Network Open.*

eTable. Menu Design for Eight Adult Patients

Menus included a total of 222 food items, which were subsequently categorized into 10 food groups: Grains (22 food items), Dairy (19 food items), Vegetables (71 food items), Fruits (20 food items), Fats/Oil, and Nuts (34 food items), Poultry/Meat (19 food items), Fish and Seafood (9 food items), Eggs (10 food items), Legumes (7 food items), and Condiments (11 food items)

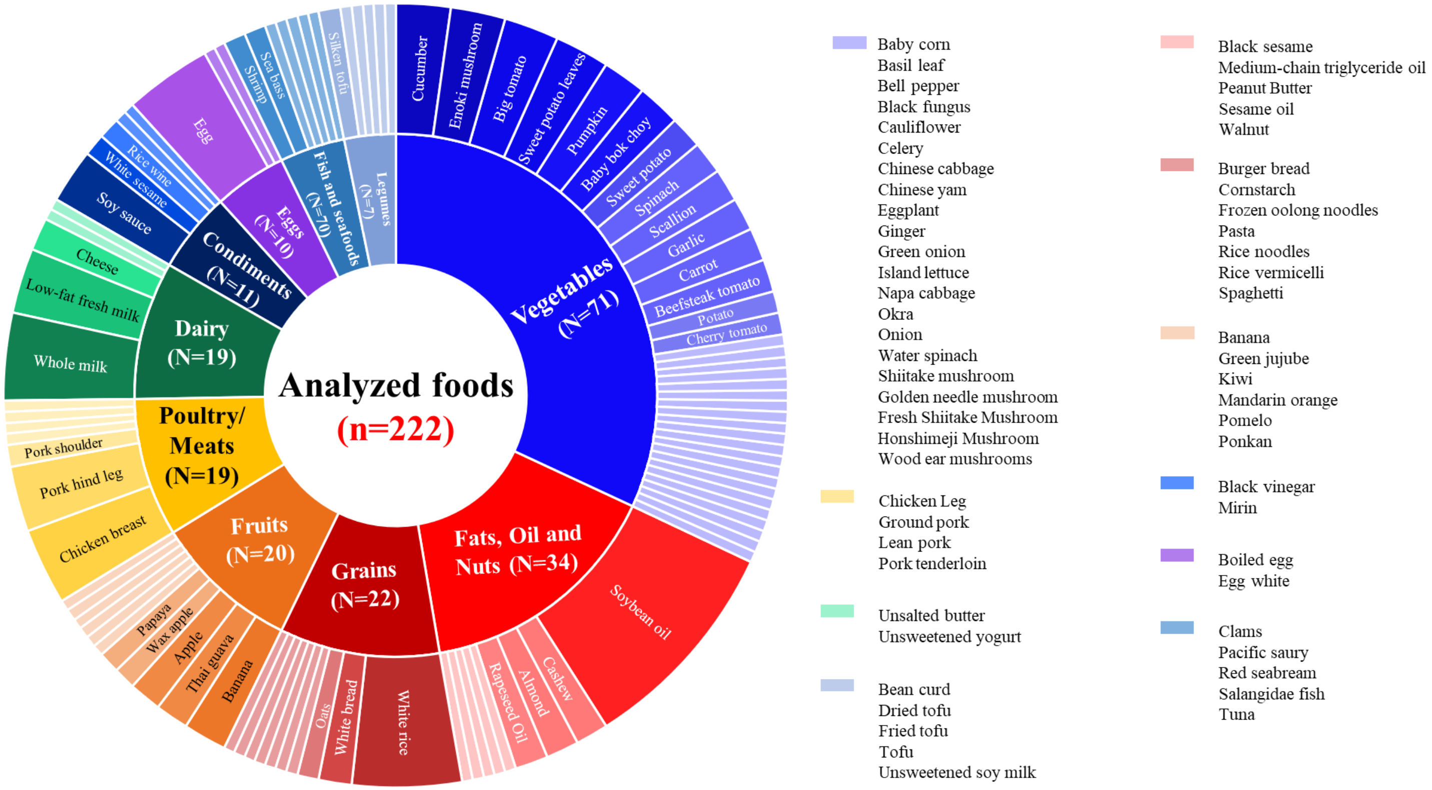

© 2023 Hoang YN et al. *JAMA Network Open.*

Case	Breakfast	Morning Snack	Lunch	Afternoon Snack	Dinner	Evening Snack
**Case 1**	Whole milk 240ml<br>Chicken breast 30g<br>Sweet potato 55g<br>Cherry tomato 110g<br>Sweet potato leaves 75g<br>Cashew 3g<br>Soybean oil 2.5ml<br>Salt 0.5g	Egg 55g<br>Salt 0.5g	Hamburger 50g<br>Pork ham 35g<br>Big tomato 40g<br>Cucumber 30g<br>White sesame 4g<br>Salt 0.5g<br>Tofu 80g<br>Carrot 30g<br>Soybean oil 2.5ml<br>Soy sauce 10ml<br>Sweet potato leaves 75g<br>Thai guava 80g	Banana 70g	Pasta 40g<br>Island lettuce 70g<br>Lean pork 35g<br>Big tomato 30g<br>Soybean oil 2.5ml<br>Salt 1g<br>Thai guava 80g	Whole milk 240ml<br>Cashews 3g
**Case 2**	Papaya 75g<br>Low-fat fresh milk 240ml<br>Chicken egg 55g<br>Salt 0.2g<br>Spinach 50g<br>Salt 0.3g<br>White rice 40g	Black sesame seeds 10g<br>Low-fat fresh milk 240ml	Thai guava 160g<br>Frozen oolong noodles 120g<br>Salangidae fish 80g<br>Tomatoes 80g<br>Spinach 20g<br>Salt 1g<br>Pork ham 52.5g	Sea bass 70g<br>Baby bok choy 40g<br>Young ginger 5g<br>Scallion 5g<br>Salt 0.5g<br>White pepper powder 0.2g	Silken tofu 70g<br>White rice 80g<br>Shrimp 50g<br>Enoki mushrooms 40g<br>Salt 0.5g<br>Scallion 5g<br>Sweet potato leaves 55g<br>Salt 0.3g	Apple 97.5g<br>Low-fat fresh milk 240ml

© 2023 Hoang YN et al. *JAMA Network Open.*

**Case 3**	Pumpkin 90g<br>Low-fat fresh milk 240ml<br>Chinese yam 90g<br>Pork ham 80g<br>Rice wine 15ml<br>Salt 3g	Cherry tomato 110g<br>Egg white 65g<br>Shrimp 50g<br>Bean curd 15g	Sea bass 70g<br>Ginger 12g<br>Eggplant 72g<br>Garlic 24g<br>Soybean oil 10ml<br>Soy sauce 5ml<br>Baby bok choy 80g<br>Carrot 22g	Skinless chicken breast 130g<br>Walnut 4g<br>Ponkan 95g<br>White rice 100g	Pork tenderloin 35g<br>Cucumber 100g<br>White pepper 0.1g<br>Cheese 22.5g<br>Black pepper 0.3g<br>Large tomato 20g<br>Chinese cabbage 32g<br>Green onion 18g<br>Wax apple 97.5g	
**Case 4**	White bread 90g<br>Tomato 90g<br>Scallion 10g<br>Egg 55g<br>Pork ham 70g<br>Soybean oil 6ml<br>Unsweetened soy milk 190ml	Fresh milk 240g<br>Almond 7g	White rice 80g<br>Sweet potato 55g<br>Dried soybeans 70g<br>Basil 5g<br>Ginger 5g<br>Sesame oil 5ml<br>Ground pork 35g<br>Napa cabbage 50g<br>Shiitake mushroom 10g<br>Bok choy 30g<br>Soybean oil 5ml	Papaya 165g	Spaghetti 50g<br>Basil leaf 10g<br>Garlic 10g<br>Soybean oil 5ml<br>Cauliflower 50g<br>Bell pepper 30g<br>Chicken breast 70g<br>Soybean oil 1ml<br>Pumpkin 85g<br>Milk 240ml	Banana 70g

© 2023 Hoang YN et al. *JAMA Network Open.*

**Case 5**	Rice vermicelli 20g<br>Cornstarch 3.5g<br>Black fungus 25g<br>Red onion 5g<br>Carrot 30g<br>Scallion 10g<br>Black vinegar 5g<br>Okra 30g<br>Cheese 22.5g<br>Egg 55g<br>Soybean oil 10ml	Banana 35g<br>Almonds 7g<br>Whole milk 120ml<br>Pumpkin 85g	Rice 50g<br>Pork ham 35g<br>Enoki mushroom 20g<br>Beefsteak tomato 30g<br>Egg 13g<br>Soybean oil 5ml<br>Baby bok choy 40g<br>Soybean oil 5ml<br>Chinese chives 10g<br>Fried tofu 35g<br>Soybean oil 2.5ml	Mandarin orange 150g	Unsalted butter 2.5g<br>Potato 45g<br>Rice 30g<br>Chicken breast 30g<br>Beefsteak tomato 30g<br>Soy sauce 5ml<br>Soybean oil 5ml<br>White sesame seeds 3g<br>Wood ear mushrooms 20g<br>Cucumber 10g<br>Soybean oil 2.5ml<br>Spinach 30g<br>Soybean oil 5ml<br>Green jujube 70g<br>Tuna 25g<br>Whole milk 240ml<br>Oats 20g<br>Cucumber 10g	
**Case 6**	Sweet potato 55g<br>Whole wheat bread 120g<br>Romaine lettuce 20g<br>Beefsteak tomato 30g<br>Almond 7g<br>Milk 240ml	Java apple 165g	White rice 160g<br>Chicken egg 55g<br>Red seabream 70g<br>Baby bok choy 40g<br>Enoki mushroom 30g<br>Unsweetened yogurt 210g	Apple 115g	Onion 50g<br>Chicken breast 60g<br>Silken tofu 140g<br>Sweet potato leaves 90g<br>Salt 4g<br>Rice wine 3ml<br>Soybean oil 15ml<br>Soy sauce 16ml<br>Green onion 25g<br>Garlic 10g<br>Fresh ginger 5g	

© 2023 Hoang YN et al. *JAMA Network Open.*

**Case 7**	White Toast 60g<br>Chicken Egg 27.5g<br>Whole Milk 48ml<br>Banana 50g<br>Soybean Oil 5ml<br>Baby corn 50g<br>Cucumber 30g	Peanut Butter 9g<br>Whole Milk 192ml	White Rice 40g<br>Pumpkin 42.5g<br>Fresh Shiitake Mushroom 20g<br>Water spinach 70g<br>Rapeseed Oil 5ml<br>Enoki Mushroom 30g<br>Pork ham 70g<br>Rapeseed Oil 5g<br>Mirin 5g<br>Kiwi 105g	Potato 90g<br>Skinless Chicken Breast 30g<br>Cheese, Shredded 17.5g	White Rice 40g<br>Chicken Leg 90g<br>Cashews 10g<br>Soybean Oil 2.5ml<br>Soy Sauce 8g<br>Chicken Egg 27.5g<br>Spinach 90g<br>Honshimeji Mushroom 10g<br>Rapeseed Oil 2.5ml	Pomelo 75g
**Case 8**	Sliced white bread 30g<br>Peanut butter 9g	Fresh milk 240ml<br>Boiled egg 55g	Pacific saury 70g<br>Salt 0.5g<br>White rice 80g<br>Sweet potato leaves 100g<br>Medium-chain triglyceride oil 2.5ml<br>Golden needle mushroom 50g<br>Soybean oil 5ml<br>Salt 0.5g<br>Pork shoulder 35g	Banana 70g<br>Skinless chicken breast 30g	Rice noodles 40g<br>Enoki mushroom 100g<br>Celery 50g<br>Soybean oil 5ml<br>Pork shoulder 70g<br>Clams 160g<br>Old ginger 1g	Apples 130g<br>Oats 40g

© 2023 Hoang YN et al. *JAMA Network Open.*
