# Supplementary material for: Consistency and Accuracy of Artificial Intelligence for Providing Nutritional Information
Source: JAMA Netw Open. 2023 Dec 27;6(12):e2350367. doi: 10.1001/jamanetworkopen.2023.50367 (PMC10753390; doi:10.1001/jamanetworkopen.2023.50367)
Supplement: Supplement 2. — Data Sharing Statement [file jamanetwopen-e2350367-s002.pdf]

Data Sharing Statement

Hoang. Consistency and Accuracy of Artificial Intelligence for Providing Nutritional Information. *JAMA Netw Open*. Published December 27, 2023. doi:10.1001/jamanetworkopen.2023.50367

Data
Data available: No

Additional Information
Explanation for why data not available: The data is currently unavailable for sharing, but we can provide it if required.
